# Multicomponent Reactions

**DOI:** 10.3390/molecules24132372

**Published:** 2019-06-27

**Authors:** Cristina Cimarelli

**Affiliations:** School of Sciences and Technology, Chemistry Division, University of Camerino, Via S. Agostino 1, 62032 Camerino (MC), Italy; cristina.cimarelli@unicam.it; Tel.: +39-0737-402241

**Keywords:** Multicomponent Reactions, small molecules, heterocycle synthesis, green chemistry, synthetic methodologies

## Abstract

Multicomponent Reactions appear to be ideal for any form of synthesis, because of their numerous advantages in terms of sustainability and selectivity in building up complex molecular architectures, with high molecular diversity. This Special Issue collects seven contributions which expand our knowledge about Multicomponent Reactions, providing a good overview about innovative reactivities and applications.

The high atom economy and bond-forming efficiency, the low-cost separation and purification of products, and the scarce waste formation make Multicomponent Reactions ideal for any form of synthesis. They offer to organic chemists the possibility to produce molecular diversity in combinatorial synthesis, and, in many cases, may be adapted to automation. The plethora of chemical reactions involved in Multicomponent Reactions adds the possibility to obtain many different molecular skeletons through beautiful and interesting chemistry. For these reasons a Special Issue collecting research on some aspects of Multicomponent Reactions represents a good opportunity for dissemination of recent progresses in this field.

1,4-Naphthoquinone scaffolds possess at the same time a changeable molecular architecture and interesting biological activities, features that make them challenging for synthetic chemists. Liqiang Wu and coworkers [1] contributes with a simple and mild synthesis of 1,4-naphthoquinones possessing indole scaffolds by the reaction of 2-hydroxy-1,4-naphthoquinone-substituted salicylic aldehydes and indoles with In(OTf)_3_ catalysis. Furthermore, substitution on salyciladehydes and indoles allows to obtain a high degree of molecular diversity.

The second contribution of Liqiang Wu and coworkers [2] deals with the synthesis of a series of spirooxindole-*O*-naphthoquinone-tetrazolo[1,5-*a*]pyrimidine hybrids. The naphthoquinone core is an active pharmacophore used in medicinal chemistry and drug discovery research. This synthesis was inspired both by the limited number of natural *O*-quinones and by the interest in the combination of two or three pharmacophores on the same scaffold to create a chemical entity that is medically more effective than its individual components. Multicomponent Reactions constitute a privileged strategy for this goal.

Tetrazoles are a class of compounds interesting for their biological properties. Andrea Basso’s research group [3] describes an efficient route for large-scale synthesis of 1,5-disubstituted tetrazoles. A cyclic chiral imine substrate is converted into the target product through an Ugi-azide three-component reaction. Additional functionalities, that subsequently may be elaborated for the generation of combinatorial libraries of enantiopure heterocycles, were introduced in the final products.

Indeno[1,2-*b*]pyrroles and acenaphtho[1,2-*b*]pyrroles possess interesting biological activities, also as inhibitors of important human enzymes and receptors. A domino three-component reaction for the synthesis of these derivatives was described by Jing Wang and coworkers [4] through a green, efficient, and convenient procedure, characterized by mild reaction conditions, high yields, and operational simplicity.

Jiannan Zhao’s group [5] contributed to this Special Issue with a review covering recent developments in asymmetric A^3^ (aldehyde–alkyne–amine) coupling for the synthesis of propargylamines, a class of building blocks involved in the synthesis of several important heterocyclic scaffolds, as direct precursors or as starting materials for the preparation of key intermediates. Introduction of chiral ligands in the metal catalysed A^3^ coupling gives the chiral bias to this reaction and modifications of the ligands enabled the highly enantioselective synthesis of chiral propargylamines, used in the construction of nitrogen-containing chiral building blocks.

Multicomponent Reactions have found application also in the field of the techniques of imaging, such as Positron Emission Tomography (PET). In the article of Alexander Dömling and his group [6] the innovative approach to PET-labeled compounds introduces ^18^F at different levels in the synthesis of several small drug-like molecules as arenes, β-lactams, tetrazoles, and oxazoles, obtained through one pot convergent Multicomponent Reactions.

α-Aminophosphonate derivatives can be considered as the P-analogues of natural α-amino acids, due to the P–C–N moiety in the α-aminophosphonic skeleton, which may mean a potential biological activity. A microwave assisted and solvent-free way for the synthesis of these derivatives has been reported by Erika Bálint’s group [7] through a Kabachnik–Fields (phospha-Mannich) reaction.

In conclusion, this Special Issue contribues to highlight the multifaceted nature of Multicomponent Reactions and the growing research area in this field, describing also some applications.

I wish to thank all of the authors for their contributions to this Special Issue, the reviewers for the careful work, and Dr Zack Li and the staff members of MDPI for the editorial support.
